# Avoiding being stung or bitten – prey capture behaviors of the ant-eating Texas horned lizard (*Phrynosoma cornutum*)

**DOI:** 10.1242/bio.058453

**Published:** 2021-03-26

**Authors:** Ismene Fertschai, Wade C. Sherbrooke, Matthias Ott, Boris P. Chagnaud

**Affiliations:** 1 Institute of Biology, University of Graz, Universitätsplatz 2, 8010 Graz, Austria; 2 Southwestern Research Station, American Museum of Natural History, P.O. Box 16553, Portal, Arizona 85632, USA; 3 Institute for Anatomy, University of Tübingen, Osterbergstrasse 3, 72074 Tübingen, Germany

**Keywords:** Horned lizard, Harvester ant, Prey capture

## Abstract

Horned lizards (*Phrynosoma*) are specialized predators, including many species that primarily feed on seed harvester ants (*Pogonomyrmex*). Harvester ants have strong mandibles to husk seeds or defensively bite, and a venomous sting. Texas horned lizards possess a blood plasma factor that neutralizes harvester ant venom and produce copious mucus in the pharynx and esophagus, thus embedding and incapacitating swallowed ants. We used high-speed video recordings to investigate complexities of their lingual prey capture and handling behavior. Lizards primarily strike ants at their mesosoma (thorax plus propodeum of abdomen). They avoid the head and gaster, even if closer to the lizard, and if prey directional movement is reversed. Orientation of captured ants during retraction is with head first (rostral), thus providing initial mucus coating of the mandibles. Prey capture accuracy and precise handling illustrates the specificity of adaptations of horned lizards in avoiding harm, and the challenges lizards face when feeding on dangerous prey.

## Abstract (precis)

**Summary:** Horned lizards, primarily feeding on dangerous and venomous harvester ants, avoid being bitten or stung by striking ants mainly on the thorax, avoiding head and gaster.

## INTRODUCTION

An important aspect in the life of an animal is its choice of food and its adaptations for finding and handling food items. In many cases, dietary choices result in predator–prey interactions, where both sides evolve adaptations that increase their own fitness (Lima and Dill, 1990; Abrams, 2000; Mukherjee and Heithaus, 2013). From the predator's point of view, these adaptations should result in getting the highest energetic gain, while spending a minimum amount of handling time on a prey item (Emlen, 1966; Pyke et al., 1977; Stephens and Krebs, 1986), and minimizing the risk in handling dangerous prey (Schmidt et al., 1989; Sherbrooke and Schwenk, 2008).

Predators have evolved many strategies to avoid being harmed by dangerous prey, often manipulating them before consumption. Meerkats prey on scorpions and remove their sting before consuming them (Thornton and McAuliffe, 2006). White ibises feeding on crabs shake males fiercely, thus forcing them to autotomize their larger claw (Bildstein et al., 1989). Coatis apply a prey rolling behavior when dealing with toxic millipedes, depleting their glandular defense chemicals before consumption (Weldon et al., 2006) and bee-eaters capturing stinging insects rub their abdomen on branches to remove sting and poison (Fry, 1969). In all these cases, the prey is large enough to be manipulated in a way to remove its defense mechanism before consumption. But what to do when your prey is tiny and abundant, and none of the options mentioned are feasible?

Texas horned lizards (*Phrynosoma cornutum*; Harlan, 1825) are native to primarily arid habitats in the southwestern USA and northern Mexico (Sherbrooke, 2003). Most populations prey nearly exclusively on ants, especially on stinging and biting seed-harvester ants (*Pogonomyrmex* spp.; Pianka and Parker, 1975; Whitford and Bryant, 1979; Schwenk, 2000; Meyers and Herrel, 2005). A study investigating feeding habits of Texas horned lizards in Arizona showed that up to 99% of prey items were ants, 95% of them harvester ants (Eifler et al., 2012). Harvester ants are, however, a dangerous food item with strong, robust, and crushing mandibles (Schmidt and Schmidt, 1989). In addition, they are able to inject an insect venom known to be among the most toxic ones to vertebrates, via a potentially lethal sting, that may autotomize in some species (Hermann, 1971; Schmidt and Blum, 1978; Schmidt and Schmidt, 1989). Texas horned lizards have evolved several adaptations to the ant's venomous sting including a plasma factor that reduces venom toxicity strongly. The LD_50_ of *Pogonomyrmex maricopa* venom is about 0.12 µg/g in mice, but 162 µg/g in Texas horned lizards (Schmidt et al., 1989). The evolution of harvester-ant venom toxicity might be linked in a co-evolutionary predator­­–prey arms race with horned lizards, at least in North America where they are sympatric (Schmidt and Schmidt, 1989; Sherbrooke and Schwenk, 2008; Schmidt, 2019).

Like other iguanian lizards, horned lizards use a prehensile tongue to capture prey, with pronounced postero-ventral hyolingual retraction (Schwenk and Throckmorton, 1989; Schwenk, 2000). They perform the whole cycle of prey capture from ingestion to swallowing within one single feeding stage unique among iguanians (Sherbrooke and Schwenk, 2008), thus decreasing the time of prey capture. During retraction the tongue and hyobranchium move pronouncedly ventral, clearing the bolus from any intraoral contact as the prey item moves directly to the pharynx (Schwenk, 2000). Horned lizards do not kill ants by biting or chewing (Meyers and Herrel, 2005; Sherbrooke and Schwenk, 2008), instead they immobilize the ants with large amounts of pharyngeal mucus to avoid their direct contact with buccal and digestive tract tissues (Sherbrooke and Schwenk, 2008). This quick prey-capture technique also minimizes attention-capturing motions, and reduces time exposed to predator detection.

Horned lizards are well camouflaged by very cryptic coloration, a dorso-ventrally compressed body with shadow-disguising lateral fringe-scales, short legs and general immobility (sit-and-wait) or slow movements (Pianka and Parker, 1975; Whitford and Bryant, 1979; Huey and Pianka, 1981). Feeding on *Pogonomyrmex* and other ants may elicit aggressive ant mobbing and, once detected and attacked by mobbing ants, horned lizards quickly distance themselves from the ants (Whitford and Bryant, 1979; Rissing, 1981; Eifler et al., 2012), with the risk of attracting predators such as greater roadrunners, shrikes, raptors, canid or felid mammals, or snakes (Sherbrooke, 2013).

Horned lizards utilize accommodation and other visual factors to judge the correct time, distance and direction of tongue protrusion during ant capture (Ott et al., 2004). Tongue protrusion trajectory is not fixed once initiated, as in some anurans (Nishikawa, 2000). Rather Texas horned lizards are capable of very rapidly (in a few milliseconds) changing the head direction and the trajectory of the tongue based on visual input from the prey (Ott et al., 2004). This behavioral plasticity may be necessary for rapid, successful capture of small, dangerous, fast-moving prey.

The aim of the present study was to further investigate lingual prey capture in horned lizards when feeding on harvester ants. We videotaped Texas horned lizards capturing harvester ants using a high-speed video system and analyzed the precision of their strike by measuring the position of the tongue's capture point relative to the ant's body length at the time of first contact. We show that strikes are aimed at the dorsal mesosomal part (between head and gaster) of the body in order to subsequently turn the ant upside down, during tongue retraction, thus avoiding being stung or bitten by the ants prior to head-first mucous embedding during swallowing. We conclude that a highly specialized myrmecophagous diet in many horned lizards has been accompanied by multiple complementary adaptations involving their blood physiology, visual integration with challenging millisecond prey capture, precise kinematics of lingual prey ingestion, and secretion of digestive tract coatings to entangle dangerous prey.

## RESULTS

### Prey-capture speed and bolus orientation

**Fig. 1.**
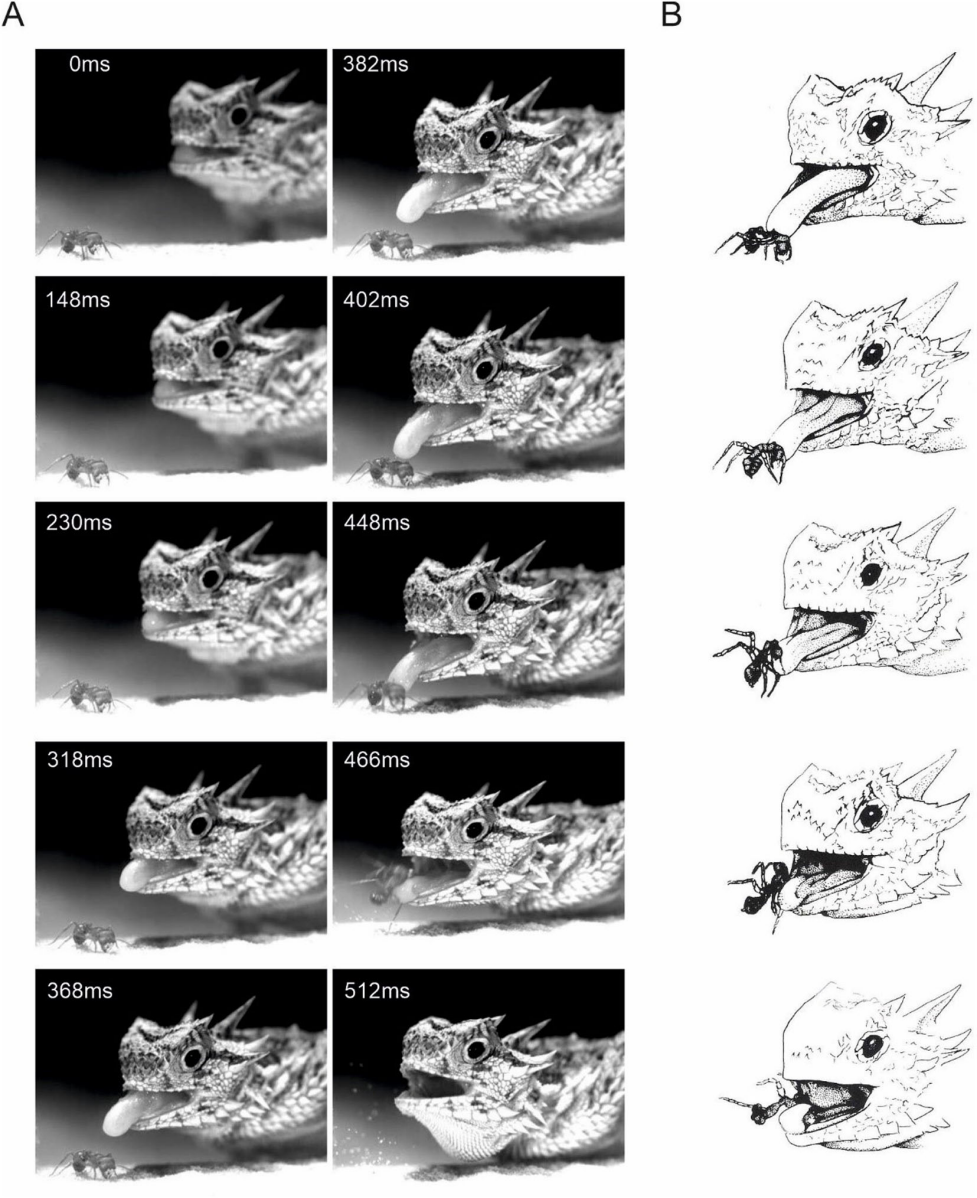
**Typical ant capture event in *Phrynosoma cornutum*.** (A) Selected video frames from a feeding sequence filmed from an anterolateral view (see Movie 2 for all images). Time (ms) is given in the upper left of each frame. The tongue of the lizard contacts the ant (*Pogonomyrmex spp.*) and is then retracted while the ant is rotated so that it comes to lie ventral side up as it is moved into the mouth, through the pharynx and swallowed during deep hyobranchial depression. (B) Schematic drawing of an ant during capture and retraction.

Horned lizards protruded their tongue while moving forward, they hit the free moving ant with the dorsal part of the fore-tongue, pushed the ant to the substrate and retracted the tongue in a rolling motion that in most cases flipped the prey around the end of the tongue in conveyor-belt fashion into the lizard's mouth. This turned the ant upside down with its legs freely extended upward into the air, and its stinger and mandibles also angled upward above the tongue's surface (Fig. 1, see Movies 1 and 2 for full sequence and pushing to substrate).

The duration of tongue protrusion (onset of tongue protrusion until the tongue hits the ant), the duration between first prey contact and retraction, as well as the duration of tongue retraction, were calculated for 17 out of 50 trials. The number differs from the absolute number of trials due to technical limitations with high-speed recording (the tongue was already protruded at the beginning or the recording stopped before the tongue was fully retracted). The duration of tongue protrusion was 60.1 ms (s.d. 13.1 ms), followed by 5 ms (s.d. 1.5 ms) from the first prey contact until retraction began, and then by 15.74 ms (s.d. 1.9 ms) for retraction.

Fig. 1B illustrates the position and orientation of the ant prey during initial tongue contact with its papillary cushion and during its retrieval, with the inverted ant advancing head forward and legs in the air. Of 46 such captures recorded, 43 showed the ant retracted head-first, in three the ant was oriented crosswise, the remaining four trials could not be analyzed due to incomplete recordings.

### Prey capture targeting behaviours

**Fig. 2.**
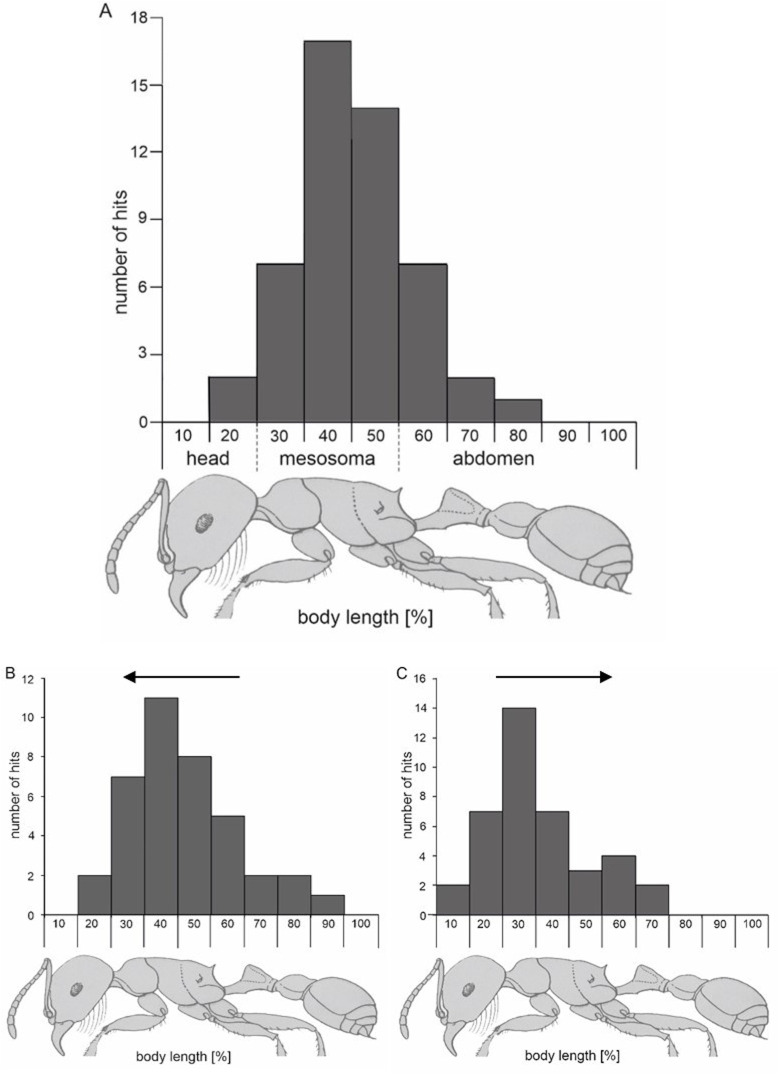
**Distribution of the lizard tongue (*Phrynosoma cornutum*) contact position on the ant body (*Pogonomyrmex spp*.).** (A) Tongue contact position in the freely moving ant condition. The relative contact position is expressed as percentage of ant body length. The distribution reflects a total of 50 trials (five lizards). (B,C) Tongue contact position on the ant body of passively moved ants, rostro-caudal (Fig. 2B) or a caudo-rostral (Fig. 2C) direction, as indicated by the arrows. The distribution reflects a total number of 38 and 39 trials, respectively (B and C, six lizards).

The contact point of the tongue in 50 strikes (five lizards) was measured as a percentage of ant body length. Unexpectedly, the tongue contact position was not aimed at the middle of the ant's body, instead, it was found to be mainly in the first 21–50% (76% of all hits) of the ant's body, (mean=40.16% (s.d.11.4); Fig. 2A; *n*=50 hits). The lizard tongue never touched the ants at their rostral or caudal end, even if it was the closest position to strike at. Thus, the lizards preferred extending their tongue further, rather than touching the ants at their closest, mandible and stinger, armed positions. For statistical analysis the ant body was divided into its main parts, head (0–20%), mesosoma (21–50%) and ‘abdomen’ (waist+gaster; 51–100%). We compared the observed strikes with evenly distributed expected strikes using the Chi² test. The number of hits at the head (adjusted standardized residuals r=−2.5) and the abdomen (r=−3.1) were significantly lower than the expected values and the number of hits at the mesosoma (r=4.6) were significantly higher than the expected value (χ² Pearson=21.743; d.f.: 2; *P*<0.001).

In staged forward and backward movement of tubed ants, horned lizard tongues’ trajectory at ants struck tubing mainly in the first 21–50% of the ant's body (Movie 3), irrespective of the apparent direction of movement [(68.4% and 71.8% of strikes for forward and backward moved ants, respectively; mean=41.25% (s.d. 16.51%) and 41.31% (s.d. 15.19%)]. We did not observe a significant difference (K-S test normality distribution was met; two tailed *t*-test: *P*=0.987) between the striking point of the lizard's tongue between these two conditions (Fig. 2B,C), suggesting that they identified the ants’ body segments and actively targeted the preferred impact points instead of just predicting the strike point from the ant's direction of moving.

## DISCUSSION

In this lingual prey-capture study, we show that horned lizards are highly selective where to contact dangerous prey items with their tongue.

Similar to earlier findings (Schwenk and Throckmorton, 1989; Meyers and Herrel, 2005), we show that the protrusion of the tongue took longer than tongue retraction. This longer time to protrude the tongue might be due to the fact that the extrusion of their tongue does not follow a fixed-action pattern, but is guided by visual accommodation capable of adjusting the direction of tongue protrusion by turning their head and/or tongue (Ott et al., 2004). This behavioral plasticity seems to be an adaptive requisite in the rapid and successful capture of dangerous, fast-moving small prey items. A recent study on a non-iguanian lizard (*Tiliqua scincoides*), that has an omnivorous diet, interestingly showed, that tongue retraction was variable and usually longer than tongue protrusion (Hewes and Schwenk, 2021).

The lizards strike ants primarily in the first 21–50% of their body (Figs 1 and 2), thereby restricting tongue contact mostly to the dorsal part of the mesosoma, which prevents the ant from flexing either the head or gaster far enough to bite or sting the lizard's tongue, and keeps their legs free. They manage this precise strike even though ants are moving fast on the ground or under experimental conditions where ants are artificially moved forwards and backwards within a tube (Fig. 2, Movie 3). Furthermore, the latter suggests that the lizards specifically target identified body parts rather than extrapolating the best-strike location from the ant's velocity vector. We suggest that during rapid tongue retraction (Movies 1, 2), this accuracy also facilitates the rotation of the ant's body towards head-first entry and swallowing due to the initial inertia of the untargeted abdomen. The precise targeting of the ant's mesosoma during prey capture in *P. cornutum* suggests strong selection on visual coordination of the lingual feeding system in order to avoid injury from dangerous prey.

**Fig. 3.**
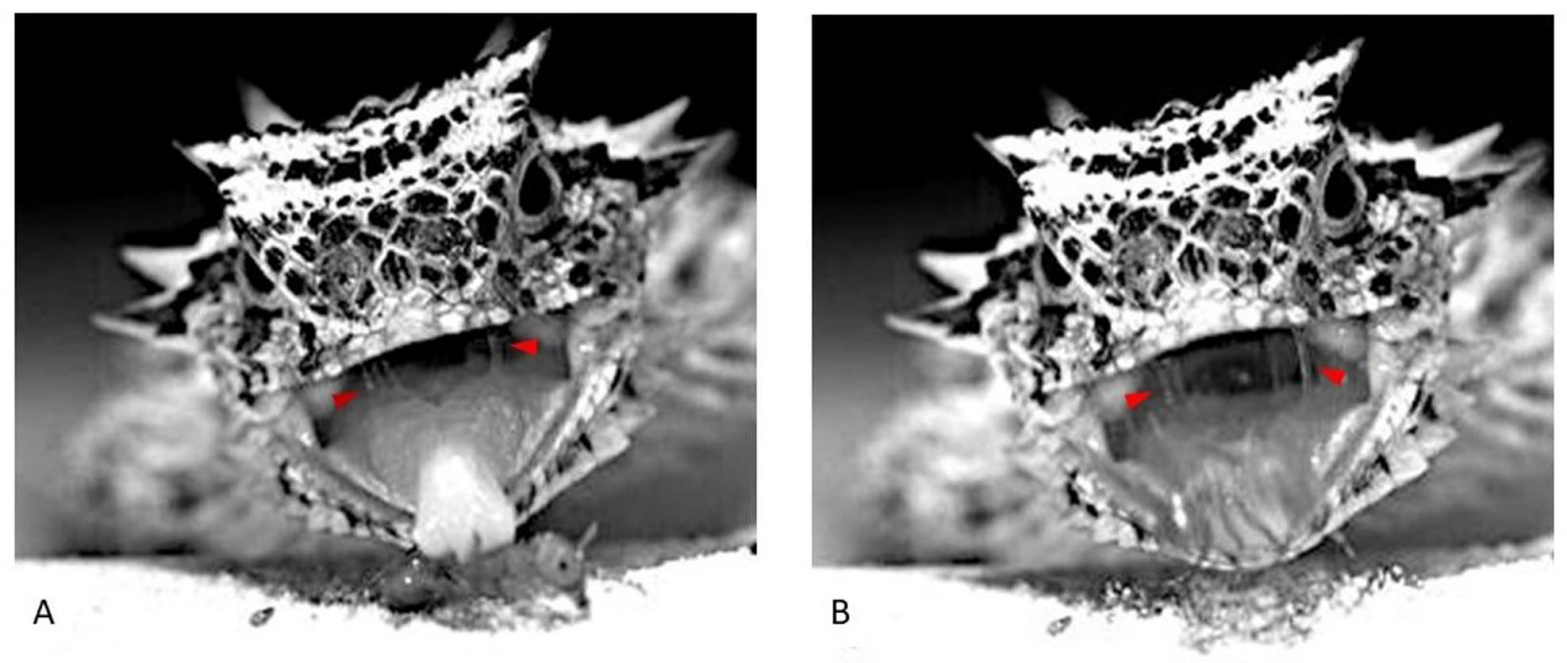
**Images of a typical harvester-ant capture by *Phrynosoma cornutum* highlighting the mucus strands.** (A) Lizard's tongue striking the ant's mesosoma with the extended tongue's dorsal papillary cushion. (B) Ant retrieval involves two independent accelerating components, that of the retraction of the papillary cushion on the tongue and that of the hyolingual retraction of the tongue. Small red arrowheads (A and B) point to mucus strands in the buccal cavity of the feeding lizard, and the blurred image of the ant (B) upon initial retrieval ‘captures’ its speed.

The rapid capture, precise targeting, head-first orientation (this study), and immediate encapsulation of *Pogonomyrmex* ant prey in mucus during swallowing (Sherbrooke and Schwenk, 2008) are apparent adaptations for consuming dangerous prey. The danger of harvester ants to their horned lizard predators is greatly exacerbated by the possibility of mobbing by other colony foraging ants (Rissing, 1981). *Pogonomyrmex* ants have mandibular glands that produce and release an alarm pheromone, 4-methyl-3-heptanone (McGurk et al., 1966; Schmidt and Schmidt, 1989). Given the almost immediate application of copious mucus (Fig. 3) to the advancing head of seed-harvester ants by horned lizards (Sherbrooke and Schwenk, 2008), we suggest a possible additional role for the mucus coating: it may eliminate or delay chemical communication between captured ants and their nest-mate column of foragers, impeding a mobbing attack of the feeding lizard (Benthuysen and Blum, 1974; Rissing, 1981).

In this study, we demonstrate that Texas horned lizards precisely target their harvester ant prey in a way that minimizes their exposure to bites and stings, and possibly to mobbing by other ants. This finding adds to a growing list of putative adaptations that enable these horned lizards, and perhaps other species, to consume large numbers of exceptionally dangerous prey: modulation of tongue trajectory during capture (Ott et al., 2004), incapacitation of ant weapons by mucus-binding (Sherbrooke and Schwenk, 2008), reduction in handling time through rapid tongue retraction and elimination of separate processing, transport and swallowing cycles (this study; Sherbrooke and Schwenk, 2008), and the evolution of a blood factor that significantly reduces the toxicity of harvester ant venom (Schmidt et al., 1989). Collectively, these characteristics of horned lizards reflect adaptive specialization for a diet that has required multiple-system adaptations to overcome the diverse and dangerous defences of harvester ants.

## MATERIALS AND METHODS

### Animals and experimental set-up

Texas horned lizards, *Phrynosoma cornutum* and harvester ants *Pogonomyrmex rugosus* and *P. barbatus*, were collected in south-western New Mexico (Sherbrooke, 2002) and were shipped to Germany (M. Ott; University of Tübingen). A scientific collection permit (#1149) was issued by the New Mexico Department of Game and Fish, and exportation was facilitated by the United States Fish and Wildlife Service, for lizards and sterile worker ants. They were housed (12 h:12 h dark:light cycle) in terraria at the University of Bonn (May 2005) and were fed diverse local ants and juvenile field crickets (*Gryllus bimaculatus*). During experiments, lizards were fed seed-harvester ants, *Pogonomyrmex* spp.. In order to analyse prey capture behavior, individual lizards were transferred to an experimental arena (glass terrarium, 60 cm/30 cm/30 cm) with a sand-covered bottom and allowed to acclimatize for 30 min. Illumination was provided by laboratory fluorescent lighting, and infrared lighting facilitated thermoregulatory behaviors. During each experiment, there was only one lizard in the arena. Immediately after animals were transferred to the arena they readily started to feed on ants that were allowed to escape from a sand-buried container through a vertical tube. Lizards readily approached the exit of the tube once they had identified it as the location of appearing ants. This localized emergence of ants allowed us to anticipate and adjust camera focusing. Additional light sources enhanced high-speed imaging.

To test if horned lizards recognized the anterior portion of the ant (their body is asymmetrical, see Fig. 2A), we placed single ants in a plastic tube (see-through plastic; diameter 0.6 cm) that was manually moved back and forth, thus generating live ants that moved forward or backwards. Dead ants were not eaten by the lizards, and preliminary experiments with dead ants being moved passively in the tube, only occasionally lead to capture attempts. Lizards struck at the moving ants but could only touch the tube. In order to keep the lizards motivated they were rewarded, after a few strikes towards the ant/tube, with live ants being dropped in front of them. In total, eight horned lizards were used in 50 free-moving ant experiments and 77 tubed-ant experiments.

### Imaging

Prey snapping was recorded with a high-speed video system (LaVision High Speed Star 4, LaVision GmbH, Göttingen, Germany) at different frame rates (250, 500, 1000 and 2000 fps). We used either one or two cameras in the experiments. One camera was always positioned to film the x–y plane of the terrarium while the additional synchronized camera was positioned above the terrarium (x–z plane). Images were exported via the camera software (DaVis 7, LaVision) as JPG files and image sequences were analyzed with custom written software (VidAna by Michael Hofmann).

### Analysis

In the free-moving ant experiments, images of prey capture in which the tongue of the lizard touched the ant were used for analysis. Relative ants’ size was measured (in pixels) and the position of the tongue on the ant was recorded in relation to the length (head to tail) of the ant. This analysis was performed for both visualization planes. The sample size varies between both planes, as the rostro–caudal axis of the ants could be perpendicular to the x–y plane, the lizard was out of the visualization area, or images were not in focus. In all but three cases, data from the camera above were used for further statistical analysis. In the remaining three cases data from the lateral view were analyzed due to distorted or unfocused images. In the experiment with the ants positioned inside the tube the point at which the tongue first touched the tube was extended towards the ant in a straight line (extrapolation) and this position was taken as the point of contact. Prey capture duration was analyzed by single frame analysis. Statistical analysis was performed with IBM SPSS Statistics.

## Supplementary Material

Supplementary information
