# Supplementary material for: Avoiding being stung or bitten – prey capture behaviors of the ant-eating Texas horned lizard (Phrynosoma cornutum)
Source: Biol Open. 2021 Mar 26;10(3):bio058453. doi: 10.1242/bio.058453 (PMC8015224; doi:10.1242/bio.058453)
Supplement: Supplementary information [file biolopen-10-058453-s1.pdf]

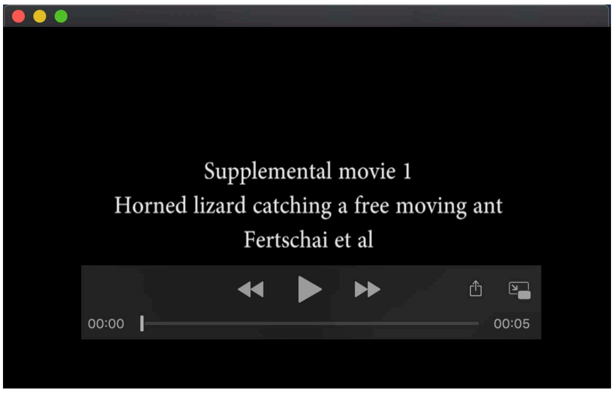

Movie 1. Movie animation of a typical prey capture trial showing a lizard and a free moving ant. Movie was slowed down by a factor of 2 (1000fps), a factor of 10 (200fps) and a factor of 100 (20fps).

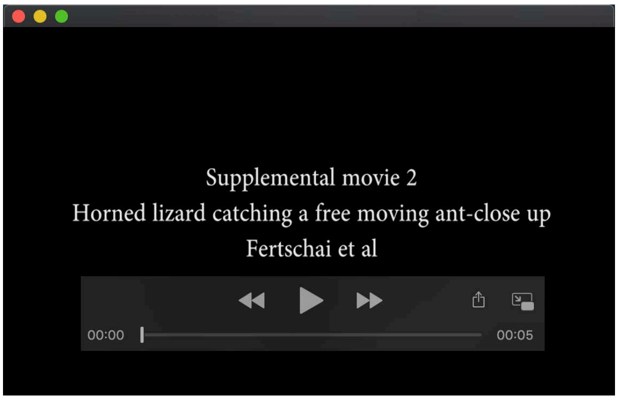

Movie 2. Movie animation of the full image sequence of Fig.1. Movie was slowed down by a factor of 20 (100fps) and a factor of 100 (20fps).

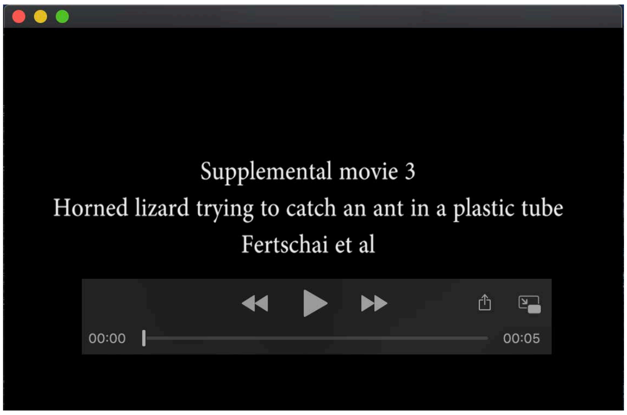

Movie 3. Movie animation of a capture trial with the ant represented in a tube. Movie in real time (250fps) and slowed down by a factor of 10 (25fps).

Biology Open • Supplementary information
